# Advancements in chondrocyte 3-dimensional embedded culture: Implications for tissue engineering and regenerative medicine

**DOI:** 10.1016/j.bj.2024.100786

**Published:** 2024-09-03

**Authors:** Yu-Ying Chu, Atsuhiko Hikita, Yukiyo Asawa, Kazuto Hoshi

**Affiliations:** a Department of Sensory and Motor System Medicine, Graduate School of Medicine, The University of Tokyo, Tokyo, Japan; b Department of Tissue Engineering, The University of Tokyo Hospital, Tokyo, Japan; c Department of Plastic and Reconstructive Surgery, Craniofacial Research Centre, Chang Gung Memorial Hospital at Linko, College of Medicine, Chang Gung University, Taoyuan, Taiwan

## Abstract

Cartilage repair necessitates regenerative medicine because of the unreliable healing mechanism of cartilage. To yield a sufficient number of cells for transplantation, chondrocytes must be expanded in culture. However, in 2D culture, chondrocytes tend to lose their distinctive phenotypes and functionalities after serial passage, thereby limiting their efficacy for tissue engineering purposes.

The mechanism of dedifferentiation in 2D culture can be attributed to various factors, including abnormal nuclear strength, stress-induced mitochondrial impairment, chromatin remodeling, ERK-1/2 and the p38/mitogen-activated protein kinase (MAPK) signaling pathway. These mechanisms collectively contribute to the loss of chondrocyte phenotype and reduced production of cartilage-specific extracellular matrix (ECM) components.

Chondrocyte 3D culture methods have emerged as promising solutions to prevent dedifferentiation. Techniques, such as scaffold-based culture and scaffold-free approaches, provide chondrocytes with a more physiologically relevant environment, promoting their differentiation and matrix synthesis. These methods have been used in cartilage tissue engineering to create engineered cartilage constructs for transplantation and joint repair.

However, chondrocyte 3D culture still has limitations, such as low viability and proliferation rate, and also difficulties in passage under 3D condition. These indicate challenges of obtaining a sufficient number of chondrocytes for large-scale tissue production. To address these issues, ongoing studies of many research groups have been focusing on refining culture conditions, optimizing scaffold materials, and exploring novel cell sources such as stem cells to enhance the quality and quantity of engineered cartilage tissues.

Although obstacles remain, continuous endeavors to enhance culture techniques and overcome limitations offer a promising outlook for the advancement of more efficient strategies for cartilage regeneration.

## 1 Introduction

### 1.1 Brief overview of chondrocytes and their role in cartilage

The cartilage is a robust and pliable connective tissue that imparts form, reinforcement, and organization to various tissues. It comprises chondrocytes, the specialized cells [1] which are responsible for the synthesis and degradation of the extracellular matrix (ECM). The ECM is characterized by a high concentration of type II collagen, proteoglycans, and other macromolecules [2] that plays important roles in maintaining cartilage integrity. Chondrocytes secrete bioactive molecules that contribute to the strength and flexibility of cartilage in a state of homeostasis by regulating its composition and organization[1], [3], [4], [5].Aging process or excessive use of cartilage results in the catabolic and aberrant differentiation of chondrocytes, causing the depletion of cartilage ECM and consequent development of osteoarthritis [6]. In contrast to other self-repairing tissues, such as bone, cartilage has a limited regenerative capacity, rendering it more challenging to self-heal following injury [7]. Consequently, to culture and differentiate the chondrocytes efficiently has become the subject of increasing research interest, not only for the cell-based therapy of osteoarthritis, but also for implantation and reconstructive procedures aimed at restoring cartilage function in various parts of the body.

### 1.2 Importance of effective chondrocyte culture for tissue engineering

For an extended period, the cartilage has been a primary area of focus in the field of tissue engineering because of the ever-increasing demand for joint repair and cartilage framework reconstruction, such as rhinoplasty or otoplasty. This concept is to “replace like with like”. However, cartilage tissue engineering requires a substantial quantity of chondrocytes to produce an adequate amount of tissue for transplantation [8], and moreover autologous chondrocytes are preferred for tissue engineering to avoid immune rejection. Most studies have used chondrocytes derived from immature animals. These cells present a higher rate of proliferation and possess an enhanced capacity for chondrogenesis when compared to cells obtained from mature animals or human donors [9]. Nonetheless, the use of chondrocytes obtained from adult animals or humans is more applicable from a clinical standpoint, though it remains difficult to preserve chondrocyte phenotype and function in vitro [10]. The success of cartilage reconstruction depends largely on how well chondrocytes proliferate, produce the ECM, and differentiate into cartilage. Therefore, the development of effective methods for chondrocyte culture is imperative for the successful tissue engineering of cartilage.

## 2 Chondrocyte biology and significance in tissue engineering

### 2.1 Background and significance of chondrocyte culture techniques

To our best knowledge, numerous techniques have been developed over several decades for cell proliferation, differentiation, and identification. Each of these techniques has advantages and disadvantages. The following methods are commonly used for chondrocyte culture.

1. Monolayer culture (2D): This is a conventional approach to chondrocyte culture, wherein cells are expanded on a level surface, such as a petri dish [11] or a culture flask [12]. As the passage of time continues, the monolayer technique has gained widespread application in chondrocyte culture due to its technical ease and high success rate in achieving cultured cells and is advantageous for investigating chondrocyte proliferation and differentiation. Although monolayer culture presents certain benefits, it has several limitations. Chondrocytes may undergo dedifferentiation in monolayer culture, resulting in the loss of their initial phenotype and function [[13], [14], [15]].

Recently, temperature-responsive culture dishes have been widely applied. This method involves culturing chondrocytes in temperature-responsive culture dishes, which allows the detachment of chondrocyte sheets without the use of enzymes. The current experiments indicated that temperature-responsive culture dishes are useful for the acquisition of cultured chondrocytes, which can subsequently be employed in a clinical setting as a replacement for periosteal patches, given that these sheets can be applied without the need for a scaffold [16].This method can be used to generate chondrocyte sheets for implantation in cartilage defects. It was found that the utilization of chondrocyte sheets exhibiting a uniform cartilaginous phenotype and adhesive characteristics [16].

2. 3D culture: This method involves culturing chondrocytes in a 3D environment such as a hydrogel or scaffold. 3D cultures can better mimic the in vivo environment [17,18] of chondrocytes and facilitate the promotion of chondrogenic differentiation. The utilization of protein-coated microspheres [[19], [20], [21]], specifically polylactide (PLA) [[22], [23], [24]], is another conventional approach for the 3D cultivation of primary articular chondrocytes. 3D culture offers several advantages, including enhanced fidelity to the in vivo environment in which cell-cell and cell-extracellular environment interactions are represented by increasing the complexity of culture materials [25]. However, it also presents certain drawbacks, such as high technical demand and difficulty in serial passage.

### 2.2 Purpose and scope of the review article

The multifaceted purpose and scope of this review encompasses several significant aspects of chondrocyte culture research and its applications in the research of cartilage biology and tissue engineering. Our intention was to provide a summary of the current state of the field, emphasize the physiological relevance of 3D chondrocyte cultures, discuss cellular behavior in 2D versus 3D, promote informed decision-making in research, and contribute to the advancement of cartilage-related sciences and clinical applications.

## 3 Traditional 2D vs. 3D culture: a comparative analysis

### 3.1 Advantages and limitations of 2D culture techniques

The advantages of monolayer (2D) culture include low technical requirements, cost-effective maintenance of the cell culture, easy observation of the proliferation status, and good performance of functional tests [25]. However, several challenges are encountered when utilizing 2D culture of chondrocytes. The most critical problem is that the expansion of chondrocytes in monolayer has been reported to result in the dedifferentiation process, thereby causing a loss of their original phenotype and function [10]. In 1960s, for dispersed cell cultures, the chondrocyte were grown in or on thin plasma clots inside the Leighton tubes [27].However, in1972, monolayer culture has been done and reported that the chondrocytes encountered morphological change into fibroblastic shape [26]. Thereafter, the “dedifferentiation” was defined as a gradual decline in the expression of functional lineage markers, namely type II collagen, aggrecans (ACANs), and proteoglycan, and assume a fibroblast-like morphology, accompanied by an anomalous ECM in chondrocytes. Dessau et al. [27] also described that the plating of chondrocytes onto tissue culture dishes in 1978 and reported that the pattern of surface-associated fibronectin changed from patchy to strand-like. They found that upon spreading chondrocytes on a culture dish, an amorphous mass of chondroitin sulfate proteoglycan was deposited in the extracellular space, whereas fibronectin formed delicate intercellular strands. In the early stages of culture, type II collagen vanishes from the chondrocyte surface and remains absent from the extracellular space [11]. At present, the process of chondrocyte dedifferentiation remains an issue for cartilage regenerative therapy because when chondrocytes become dedifferentiated, the repaired cartilage they create is dysfunctional in vivo [28]. In general, 2D culture experiences alterations in cellular morphology, polarity, and mode of division, as well as an inability to replicate the inherent structures of tissues and a deficiency in depicting interactions between cells and their extracellular environment.

### 3.2 Rationale for exploring 3D embedded culture

As an enhanced approach to replace 2D culture, 3D culture presents several advantages, including greater fidelity to the in vivo environment as a result of increased complexity of culture materials, the capacity to replicate the natural structures of tissues, the representation of interactions between cells and their extracellular environment, and, most notably, a decrease in the dedifferentiation of chondrocytes when compared to 2D culture [25,29,30].

● Dedifferentiation mechanism of chondrocyte

Although the mechanism of dedifferentiation is yet to be thoroughly explored, leaving ample room for further discovery, numerous investigations have focused on the pathophysiology of this phenomenon at the cellular and nuclear levels.

1. **The abnormal nuclear strength.** During serial 2D culture passages, the flattened cellular morphology and potentially compromised nuclear envelope of the expanded chondrocytes give rise to induce an abnormal intranuclear strain. Their proposition was that this aberrant nuclear strain may constitute a significant factor that drives chondrocyte dedifferentiation towards a fibrotic cellular phenotype in monolayer culture and conceivably in mechanically challenged regenerative cartilage following implantation. Abnormal intranuclear strain and weakened nuclear envelope are fundamental constituents of the proposed mechanobiological circuit that operates in a positive feedback loop to divert chondrocytes from their native phenotype [31].
2. **The stress induced mitochondrial impairment and chromatin remodeling.** Chen and colleagues [32] al. Proposed a biphasic dedifferentiation model comprising the early and late dedifferentiation stages. In the early stages, chondrocytes display a glycolytic phenotype characterized by increased expression of genes implicated in metabolism and antioxidation. Conversely, in the later stages, chondrocytes manifest ultrastructural alterations that entail mitochondrial impairment and chromatin remodeling associated with stress.
3. **Activation of ERK-1/2 pathway**: Elevated MEK-ERK1/2 pathway activity induces chondrocyte dedifferentiation. Conversely, the use of the inhibitor (PD0325901) to suppress ERK1/2 activity reversed dedifferentiation and promoted redifferentiation. These findings provide compelling evidence that the inhibition of MEK-ERK1/2 activation can effectively impede chondrocyte dedifferentiation and fibrocartilage formation [33].
4. **Activation of the p38/mitogen-activated protein kinase (MAPK) signaling pathway**: Under typical monolayer culture conditions, p38 MAPK signaling facilitates chondrocyte differentiation. In contrast, suppression of p38 MAPK using an inhibitor (SB203580) has been observed to maintain the chondrogenic phenotype, which is a desirable outcome in the field of cartilage tissue engineering [34].

The dedifferentiation process is characterized by global inhibition of ECM synthesis and translation-related genes. This phenomenon may contribute significantly to the loss of chondrocyte function. Additionally, it has been observed that dedifferentiated chondrocytes in monolayer culture exhibit increased cell stiffness, which is attributed to a cell membrane to F-actin adhesion mechanism [31]. The state of actin polymerization has a pivotal role in the process of chondrocyte dedifferentiation [35].

### 3.3 The key differences and benefits from 3D culture

● **What are the differences in cell morphology, phenotypic stability between chondrocytes cultured in 2D and 3D**

A significant correlation exists between the characteristics of articular chondrocytes, including their shape, cytoskeleton, chondrogenic phenotype, and the metabolism of hyaline-like cartilage ECM molecules [36]. The chondrocytes present in healthy cartilage, when subjected to the 2D culturing method, undergo a transformation from their initial smooth elliptical and spheroidal shapes [37] to a fibroblastic morphology [38] characterized by flattened, elongated and spindle shapes in the later stages. On the other hand, chondrocytes cultivated in a 3D environment exhibit a morphology that is more akin to that of chondrocytes in native cartilage, characterized by a rounded shape, and tend to form spheroids or aggregates [39]. A study has demonstrated that the spherical cell morphologies, which are a distinguishing characteristic of articular chondrocytes, exhibited an increase from 84% to 99% of round cells between day one and day eight. This level was sustained throughout the extended gradient-based 3D culture [40]. It is believed that high-density 3D cultures are conducive to preserving the chondrocyte phenotype and facilitating the redifferentiation of dedifferentiated articular chondrocytes [43].In addition, the polymerization status of actin and/or tubulin also serves to modulate the chondrocyte phenotype during differentiation in 3D culture, as observed in monolayer culture. The reorganization of the cytoskeleton through actin depolymerization is deemed an active regulatory mechanism for the redifferentiation of passaged chondrocytes [35]. The paramount significance of cellular morphology in the modulation of chondrocyte phenotype is underscored, and a reversible system for the investigation of gene expression is presented [41].

● **What are the differences in cell proliferation rates and viability between chondrocytes cultured in 2D and 3D**

In rat articular chondrocytes, it appears that alginate beads (3D) create an environment conducive to proliferation and differentiation during the initial stages of the cultivation period compared to monolayer [42]. In a human study, it has been demonstrated that chondrogenic culture (induced pluripotent stem cell (iPSC)) in a 3D environment exhibits greater rates of proliferation than those grown in a 2D setting [43]. Nonetheless, a redifferentiation model of chondrocyte culture in both 2D and 3D environments demonstrated that chondrocyte proliferation was predominantly observed in 2D cultures. The highest increase in cell numbers was observed in the 2D monolayer redifferentiation cultures [30]. One study showed that when comparing the cultures of cell proliferation in 2D and 3D culture in atelocollagen gel, it was observed that the cells in the 2D culture exhibited a significantly higher level of metabolic activity during the week following chondrocytes seeding [44]. Although not specific to chondrocytes, this observation and controversies highlights the difficulty in determining the optimal culture method for cellular proliferation and differentiation, as many of the variances are dependent on the particular cell line and matrix [45]. The rate of cell proliferation is also contingent upon the specific type of 3D model in which the cells are cultivated [46].**What are the differences in cell differentiation potential, gene expression profile and ECM synthesis between chondrocytes cultured in 2D and 3D.**

There were notable differences in the differentiation potential and gene expression of chondrocytes cultured in 2D and 3D environments. It is well established that flattened fibroblast-like cells located at the periphery of cartilage colonies, commonly referred to as dedifferentiated cells, exhibit a greater propensity for synthesizing type I collagen. Conversely, round or polygonal chondrocytes typically synthesize type II collagen [47]. Specifically, chondrocytes cultured in monolayers have been observed to undergo dedifferentiation, resulting in the loss of their original round phenotype and function, expressing more collagen type X (COL10A1), matrix metalloproteinase 13 (MMP13), runt-related transcription factor 2 (RUNX2), osteopontin (OPN), vascular endothelial growth factor A (VEGFA), and alkaline phosphatase (ALP). Whereas, chondrocytes cultured in 3D have demonstrated a greater ability to maintain their phenotype and function, increased expression of ECM gene, producing higher levels of type II collagen (COL2A1), ACAN, and link protein, which are recognized markers of chondrogenic differentiation [30,48]. Additionally, chondrocytes cultured in 3D have exhibited elevated levels of SRY (sex determining region Y)-box 9 (SOX9) that governs chondrogenic differentiation, and have been observed to re-express the differentiated collagen phenotype when cultured in 3D environment [41]. Sox9 is an essential transcription factor that plays a key role in the proper progression of the chondrocyte differentiation pathway during endochondral bone formation. It was found that Sox9 and β-catenin (a key transcriptional factor in Wingless–Int (Wnt) signaling) physically and functionally interact with each other. The similarities between the phenotypes of embryos overexpressing Sox9 in chondrocytes and those in which the β-catenin alleles are inactivated in chondrocytes, and vice versa, suggest that the interactions between Sox9 and β-catenin regulate the activity of the canonical Wnt signaling pathway in chondrocytes. This regulation, in turn, mediates, at least in part, chondrocyte proliferation and differentiation [49]. With regard to the ECM, it has been observed that the molecules which exhibit binding affinity towards collagen, namely integrin subunit alpha 2 (ITGA2) and integrin subunit alpha 10 (ITGA10), demonstrate a greater degree of expression in 3D cultures utilizing atelocollagen as compared to their 2D counterparts [44]. The upregulation of members of the matrix metalloproteinase family (MMPs) occurs during the initial stages of cultivation in the presence of atelocollagen gel when comparing different 3D culture method.

These findings suggest that chondrocytes cultured in 3D have greater differentiation potential, and that the mRNA expression levels of chondrogenic differentiation-associated genes increased over time in chondrocytes cultured in 3D scaffolds compared to those cultured in a 2D environment.

● **How does chondrocytes in 3D culture interact differently with neighboring cells compared with 2D**

In 3D cultures, chondrocytes exhibit distinct interactions with neighboring cells compared to 2D cultures. Notable differences include enhanced cell-cell contact facilitated by the formation of aggregates and spheroids, which contrasts with the limited cell-cell interactions observed in 2D culture where cells are grown as a monolayer [50]. Additionally, 3D culture systems providing a more physiologically relevant environment for chondrocytes, allowing for improved cell-ECM interactions that can influence cell behavior, including cell differentiation, proliferation, and gene expression [46]. Furthermore, chondrocytes in 3D culture may display altered morphology, motility, and polarity compared to their 2D counterparts, which can have significant implications for tissue-specific functions and behaviors. Finally, in 3D cultures, chondrocytes can receive stimuli from the local environment, similar to what occurs in vivo, which can contribute to the maintenance of their differentiated phenotype and the development of more functional cartilage tissue in engineered constructs [25][Table 1].

**Table 1:** Comparison of 2D and 3D chondrocyte cultures.

Aspect	2D Chondrocyte Culture	3D Chondrocyte Culture
**Cell Growth**	Rapid cell proliferation [51]	Limited cell expansion [30,52]
		Slower cell proliferation [51]
**Extracellular Matrix**		
Advantages	Easier to study ECM by performing functional assays [52]	Enhanced ECM production and cell-ECM interactions [52]
Disadvantages	Limited ECM production [30,52]	More difficult to study ECM [52]
	Reduced ECM quality [52]	
	Inadequate cell-ECM interactions [30]	
**Cell Phenotype Maintenance**		
Advantages		Smooth elliptical and spheroidal shapes Improved cell phenotype maintenance [12,53]
Disadvantages	Flattened and fibroblastic morphology [12]. Dedifferentiation is common [12]	Still some risk of dedifferentiation [30]
**Cell-cell interactions**	Poor [25]	Good [25]
**Gene Expression**	Higher COL10A1, MMP13, RUNX2, OPN, VEGFA, and ALP expression [54]	Higher COL2A1, ACAN, SOX9, and link protein expression [55]
**Tissue Mimicry**	Limited representation of in vivo conditions [32]	Improved recapitulation of in vivo characteristics [32,56]
**Cell Passaging**	Simple to perform [25]	Difficult to perform [25]
		More challenging cell observation and measurement
**Clinical Relevance**	Limited applications due to dedifferentiation [30]	Greater clinical relevance for cartilage tissue engineering [30]
**Overall Complexity**	Simpler culture systems [25]	More complex culture systems, but with potential benefits [25]
**Cost**	Low [25]	High [25]
**Technical Requirements**	Low [25]	High, complex and resource-intensive [56] culture systems may be required [25]

## 4 Methods of chondrocyte 3D embedded culture

What are the most commonly used materials for 3D culture of chondrocytes.

### 4.1 Scaffold-based approaches

#### 4.1.1 Hydrogel scaffolds

Some of the most commonly used materials for the 3D culture of chondrocytes include.

I. Collagen hydrogels, naturally derived hydrogels, can support cell adhesion and proliferation and have been extensively employed for the 3D culture of various cell types, including chondrocytes. These hydrogels predominantly consist of type I collagen [57], although types II and III, along with additional constituents such as glycosaminoglycans, can also be integrated. Despite the controversy surrounding the ability of the type I collagen hydrogel to preserve the phenotype and function of chondrocytes in 3D culture, the findings of a study which conducted by Hu et al. [58] indicated that the mRNA expression of chondrogenic phenotypes, such as SOX9, type II collagen, and aggrecan, was significantly upregulated, particularly in the 1 mg/ml collagen gel. High concentrations of type I collagen hydrogels (7 and 5 mg/ml) also have the potential to induce chondrocyte fibrosis and enhance the expression of type I collagen, type X collagen, and Sox9 mRNA. This indicated that the presence of a high concentration of type I collagen hydrogel may lead to premature hypertrophy of articular chondrocytes. The production of type II collagen and glycosaminoglycan (GAG) content was also enhanced when culturing chondrocytes in the collagen gel [59]. Additionally, collagen type zero, sourced from jellyfish material, is also employed in conjunction with a natural crosslinker known as genipin for the purpose of chondrocyte-embedded culture [60].
II. Gelatin hydrogels, an amorphous variant of collagen possessing an identical sequence of amino acids but devoid of the triple helical structure, is also widely utilized as a hydrogel substance [61]. It can imitate the microenvironments of natural tissues and encapsulate cells homogeneously, making them an appealing option for 3D chondrocyte culture. The hydrogel scaffold composed of gelatin effectively stimulated chondrocyte proliferation, thereby facilitating the expression of collagen type II, aggrecan, and sox9. Additionally, it successfully maintained the structural stability and durability of the cartilage even under dynamic compression, ultimately promoting the process of cartilage repair [62]. It can be mixed with other ingredients, such as methacryloyl (MA), and has been reported to affect various cell behaviors, such as cell viability, cell morphology, and preservation of the chondrogenic phenotype. Gelatin methacryloyl (GelMA) hydrogels with a notable stiffness of 29.9 kPa (kPa) demonstrated superior outcomes in preserving the chondrogenic phenotype [53].
III. Hyaluronic hydrogels (HA), a biopolymer that occurs naturally in cartilage and synovial fluid, own its properties that facilitates cell adhesion and proliferation, regulates inflammation, and promotes cartilage regeneration. It has been demonstrated that augmenting stiffness through elevating the concentration of hyaluronic acid dialdehyde in the hydrogel results in the preservation of the spherical phenotype of the embedded chondrocytes, a uniform distribution within the hydrogel, and the formation of spherical aggregates. Additionally, the expression of collagen type II and glycosaminoglycans was found to be higher in stiffer hydrogels than in softer ones [63]. they have the potential to be integrated with other biomaterials, such as alginate and collagen, to produce composite hydrogels that are conducive to chondrocyte cultivation and preservation of their phenotype. The utilization of these composite hydrogels can offer a more biomimetic milieu for chondrocytes, resulting in superior tissue regeneration results [64]. There has a significant amount of research has been conducted on multifunctional hyaluronic acid-based hydrogels, which encompass a range of modifications such as alkenyl, aldehyde, thiolated, phenolized, hydrazide, and host-guest groups. These hydrogels have been extensively investigated for potential applications in cartilage tissue engineering [65]. To replicate the composition of the natural cartilage ECM, which consists of core proteins and glycosaminoglycans, a biological hydrogel was synthesized using the biopolymers HA, chondroitin sulfate (CS), and gelatin through click chemistry. Subsequent in vitro cell culture experiments demonstrated that the hydrogel effectively facilitated the adhesion and proliferation of chondrocytes [66]. Furthermore, the HA hydrogel possesses the characteristics of injectability, in situ formation, and click-crosslinking [67]. These hydrogels present the benefit of convenient administration and can be chemically altered to include bioactive substances that promote the chondrogenic differentiation of stem cells. Additionally, it has been demonstrated that HA hydrogels facilitate the development of integrated bone tissue via endochondral ossification in vivo by utilizing mesenchymal stem cells (MSCs) [68]. This underscores the immense potential of HA hydrogels not only in chondrocyte cultivation, but also in wider applications within the field of tissue engineering.
IV. Polyethylene glycol (PEG) and chondroitin sulfate (CS) hydrogels. The use of these 3D biomimetic hydrogel culture systems offers numerous advantages. It has been demonstrated to uphold and enhance chondrogenic phenotype and facilitate cell-mediated matrix degradation and turnover [55]. It has the ability to elicit zonal-specific reactions from chondrocytes, leading to the formation of zonal cartilage that closely resembles natural tissues. This sheds light on the significance of cell-matrix and cell-cell interactions in facilitating zonal development during tissue morphogenesis and regeneration [69]. The incorporation of CS, a prominent constituent found in articular cartilage, allows cells to break down the hydrogel matrix by secreting chondroitinase and depositing newly synthesized cartilage ECM [70,71]. Furthermore, CS has demonstrated anti-inflammatory properties within the arthritic joint. Biomimetic hydrogels can also serve as scaffolding materials for cell delivery in cartilage repair and can be chemically modified to enhance tissue-biomaterial integration [72,73]. Certain research endeavors have introduced interpenetrating network (IPN) hydrogels comprising agarose-poly (ethylene glycol) diacrylate (PEGDA) for cartilage tissue engineering. These hydrogels have demonstrated the ability to encapsulate viable cells and exhibit a noteworthy enhancement in mechanical performance compared to their individual constituent hydrogels [74]. The investigation also introduced a pioneering methodology for incorporating aggrecan as a bioactive stimulus for cells enclosed within IPN hydrogels utilized in the field of cartilage tissue engineering. This innovative approach results in the enhanced functionality of encapsulated chondrocytes [75].
V. Fibrin hydrogels, another naturally derived material, can be used for 3D chondrocyte culture and provide a supportive environment for cell growth. They are easily formed by the polymerization of fibrinogen and thrombin. It has demonstrated the ability to enhance chondrocyte viability, synthetic functionality, and the development of tissues exhibiting cartilage-like characteristics both within controlled laboratory environments and in living organisms. Consequently, these hydrogels hold significant potential as a material for the reparation and rejuvenation of cartilage in medical applications [75].A significant obstacle associated with fibrin-based biomaterials lies in their inadequate mechanical strength. To address this issue, scientists have attempted to enhance fibrin hydrogels by incorporating crosslinked hyaluronic acid methacrylate (HA-MA). This modification enhances the mechanical properties of biomaterials, rendering them more appropriate for chondrocyte culture and tissue engineering applications [76]. Additionally, a study has unequivocally established the superior chondrogenic properties of pure fibrin in comparison to both silk-modified fibrin and PEG-dextran [77]. Specifically, the fibrin hydrogel exhibited elevated cell viabilities and chondritic behavior, with an increase in matrix stiffness linked to the characteristic features of redifferentiation, spherical morphologies, and particularly matrix synthesis [30].
VI. Alginate hydrogels, which are naturally derived polysaccharides, can form hydrogels through ionic crosslinking and have been used for 3D chondrocyte cultures because of their biocompatibility and ability to support cell viability and matrix production. This offers a 3D microenvironment that facilitates the proliferation of chondrocytes and preserves their chondrogenic phenotypes. This development is of significant importance for chondrocyte-based therapies aimed at repairing cartilage, as it provides a material wherein cell expansion can be conducted in situ [78]. The characteristics of alginate hydrogels, including their elasticity and stability, can be regulated through the selection of alginate type, concentration, gelation method (ionic or covalent), and divalent cations as gel-inducing ions. This adjustability provides researchers with the opportunity to customize the hydrogel properties to effectively facilitate chondrocyte culture and tissue engineering [79].
VII. Agarose hydrogels, a type of 3D matrix composed of helical agarose molecules organized in supercoiled bundles, have been employed for the cultivation of chondrocytes in a 3D environment and in the field of cartilage tissue engineering owing to their biocompatibility, adjustable characteristics, and capacity to facilitate cell encapsulation. Agarose hydrogels have been integrated with additional biomaterials such as alginate to generate composite hydrogels that enhance the in vitro differentiation of stem cells into chondrocytes. This further exemplifies the adaptability and potential of agarose-based hydrogels in improving chondrocyte culture and tissue engineering applications [80]. However, the research findings indicated that agarose gels demonstrated a comparatively reduced rate of chondrocyte proliferation and GAG secretion in comparison to alginate, Matrigel, and collagen [81]. This disparity could potentially be attributed to the absence of native ligands in agarose, which are essential for facilitating interaction with mammalian cells.
VIII. Dextran-tyramine (Dex–TA) hydrogels, a type of hydrogel formed by the self-assembly of dextran and tannic acid, have been found to retain chondrocyte viability and normal morphology, making them suitable for 3D chondrocyte culture. Dex-TA hydrogels can undergo in situ gelation via a biocompatible enzymatic crosslinking reaction, forming covalent TA-TA bonds. This gelation process supports the survival and growth of incorporated chondrocytes and MSCs, as well as the deposition of a new matrix in vitro. The biocompatibility of Dex-TA hydrogels has been demonstrated by the initially high cell viability of over 90% [82]. Dex-TA hydrogels have been integrated with hyaluronan (HA) to produce Dex-TA/HA-TA hybrid hydrogels, or alternatively combined with gelation to generate HA-g-Dex-TA. These novel hydrogels exhibit considerable potential for cartilage tissue engineering. These hydrogels exhibited significant potential as injectable scaffolds in cartilage tissue engineering [83].

#### 4.1.2 Nanofiber scaffolds

Aligned nanofibrous scaffolds can replicate the inherent characteristics of the ECM, leading to the promotion of fibrochondrogenesis. The nano-fibrous architecture effectively maintained the structural integrity of chondrocytes while enhancing the expression of cartilage markers and facilitating the formation of the ECM. This attribute renders nanofiber scaffolds an appropriate medium for the adhesion, proliferation, and differentiation of chondrocytes [84].

#### 4.1.3 Decellularized matrices

The decellularized matrix is a scaffold or ECM derived from tissue that has undergone removal of its cellular constituents while preserving the essential structural and biochemical signals required for cell attachment, proliferation, and differentiation. Decellularized extracellular matrix (dECM) is a biomaterial that effectively maintains the natural environment of tissues and enhances cellular activity. This characteristic renders decellularized matrices a viable choice for chondrocyte culture, as they offer a microenvironment that closely resembles physiological conditions, surpassing the capabilities of synthetic scaffolds [85]. Chondrocytes that have been cultured on decellularized matrices have been observed to maintain their chondrogenic properties, phenotype, and function, which encompass the synthesis of ECM components specific to cartilage [86].

### 4.2 Scaffold-free approaches

Scaffold-free methodologies have been investigated in the field of 3D chondrocyte culture to harness their potential for cartilage tissue engineering. These approaches have shown promise for promoting chondrogenesis and preserving the mechanical characteristics of tissues. The absence of foreign substances, ability to generate uniform cell populations, and potential for large-scale production are among the advantages offered by scaffold-free techniques. However, it is important to note that these methods often yield tissue constructs with a higher number of chondrocytes and a lower number of ECM components than native cartilage. This discrepancy can affect the functional properties of the engineered tissue and may necessitate further refinement. Some prevalent techniques [87].

#### 4.2.1 Hanging drop method

The hanging drop method is a widely recognized technique for 3D chondrocyte culture, which employs the force of gravity to stimulate cellular aggregation and create cell spheroids. This method has been employed in numerous studies to enhance the chondrogenic commitment of isolated adult human chondrocytes and facilitate the redifferentiation of chondrocytes expanded in vitro. The combination of 3D chondrocyte cultures in hanging drops and a low-oxygen environment presents a straightforward and expedient approach for producing microstructures resembling cartilage [88].

#### 4.2.2 Spheroid culture

Spheroid culture in 3D chondrocyte culture involves the cultivation and aggregation of chondrocytes into spherical structures or clusters in a 3D environment. These structures are commonly known as “spheroids.” This technique has been extensively investigated as a 3D method for chondrocyte cultivation. This approach has several benefits including enhanced chondrogenesis, improved tissue architecture, and promising prospects for utilization in the fields of cartilage tissue engineering and regenerative medicine. The spheroid culture system has the potential to serve as a high-throughput strategy for investigating the molecular mechanisms governing chondrogenesis and initiating the initial phase of endochondral ossification [89].

#### 4.2.3 Bioreactor systems

Bioreactor systems, such as rotating-wall vessels [90] have been developed and utilized for 3D chondrocyte culture, offering controlled and dynamic environments that can enhance cell proliferation, differentiation, and ECM production. It enhances the caliber of cartilage tissue engineering by incorporating physiological environmental factors inherent in natural cartilage, including oxygen and pH levels, nutrient supply, and mechanical loading. The potential of bioreactors in tissue engineering is evident, although the impact of stimulation through diverse mechanical loads on cellular behavior remains controversy [91] [Table 2].

**Table 2:** Methods of 3D chondrocyte culture.

Categories	Method description	Advantages	Disadvantages
**Scaffold-Based Culture**	Chondrocytes are embedded within 3D scaffolds, such as hydrogels or biomaterials [86].	Provides structural support for chondrocyte growth [86].	Higher complexity [55] Higher cost [55] Limited scalability [55] Challenging to control the microenvironment [55]
		Mimics the native cartilage microenvironment [86].	
		Promotes ECM production [86].	
Hydrogel Scaffolds	Chondrocytes are cultured in a water-absorbent, cross-linked polymer networks that have a high-water content and exhibit biocompatibility and tunable properties as a support structure [55].	Tunable, simple to control mechanical properties [55]	Limited cell proliferation and differentiation [55]
		High porosity with simple cell seeding [55]	Limited cell-matrix interactions [55]
		Present versatility and biodegradability [55]	Potential of cell death [55]
Nanofiber Scaffolds	Chondrocytes are cultured in a nanoscale fiber structure [84].	Mimicry of articular cartilage nature [84]	Limited cell proliferation and differentiation [55]
		Manufacturing versatility [84]	Limited cell-matrix interactions [55]
		High porosity [84]	Limited mechanical properties [55]
		Enhanced chondrogenic differentiation [84]	Potential of cell death [55]
Decellularized Matrices	Chondrocytes are cultured in a scaffold or ECM derived from tissue, which has undergone the removal of its cellular constituents [85]	Preservation of native environment [85]	Variable matrix Composition [86]
		Biocompatibility and biodegradability [85]	Difficulties in handling [86]
		Reduced immunogenicity [85]	Less control over cell behavior [86]
		Versatility and customization [85]	Complexity of decellularization process [86]
**Scaffold-Free Approaches**	Chondrocytes cultured in a 3D milieu without foreign substances [87]	Faster remodeling and integration [87]Preservation of native cell-cell interactions [87]	Limited cell proliferation and differentiation [55]
			Uncontrolled mechanical properties [55]
			Less cell-matrix interactions [55]
			Limited assay compatibility [55]
			Less clinical application [55]
Hanging Drop Method	Chondrocytes are suspended as droplets from the lid of a culture dish [88].	Promotes self-organization of chondrocytes [88].	Difficulty in handling [29]
		Facilitates cartilage-specific ECM production [88].	
		Allows for easy manipulation of cell aggregates [88].	
Spheroid Culture	Chondrocytes are cultured and aggregated into 3D spherical structures or clusters referred to as “spheroids.” [29,89]	Simplified fabrication process [89]Preservation of native cell-cell interactions [29,89]Enhanced chondrogenic differentiation [29,89]	Diffusion gradients that lead to heterogeneity of diameter and size [29]
			Self-disassembly [29]
Bioreactor Systems	A specialized apparatus designed to facilitate the growth and maintenance of chondrocytes in a 3D environment under controlled conditions [90].	Provides dynamic culture conditions, such as perfusion and mechanical stimulation	Time-consuming and costly [90]
		Enhances chondrocyte proliferation and differentiation [90].	Limited understanding of matrix biology [90]

## 5 Overcoming challenges and future directions in 3D chondrocyte culture

### 5.1 Limitations and solutions

There are several limitations associated with 3D chondrocyte culture.

First, the complexity and potential cell viability concerns associated with certain 3D culture systems lead to significant challenges in the design and implementation of chondrocyte-based 3D culture systems [55]. Various strategies have been explored to overcome this limitation and optimize the conditions for 3D chondrocyte culture. One such strategy involves incorporating growth factors, such as transforming growth factor-beta (TGF-β), insulin-like growth factor (IGF), fibroblast growth factor (FGF), bone morphogenetic proteins (BMPs), and some signaling molecules [92] into the culture medium. TGF-β1 and IGF-1 combination promote chondrogenic differentiation, proliferation, and increase collagen and proteoglycan synthesis in 3D cultures. They have been shown to enhance redifferentiation of dedifferentiated chondrocytes and improve cartilage matrix production [93]. bFGF, TGF-β2, IGF-1, Insulin-Transferrin-Selenium (ITS), and platelet-derived growth factor (PDGF) combination has demonstrated high proliferative capacity in chondrocytes while maintaining genetic stability. ITS has several significant effects on chondrocyte culture, including enhancing proliferation, maintaining chondrogenic phenotype, improving cartilage-specific matrix production, and helping dedifferentiated chondrocytes to redifferentiate [94,95].On the other hand, PDGF activates the GIT1-PLC1-mediated ERK1/2 pathway [94], playing an important role in chondrocyte migration and survival, inhibiting apoptosis, and maintaining the chondrocyte phenotype in culture [95,96]. All of these growth factors can help amplify the limited number of chondrocytes needed for clinical applications [97] and effectively promote chondrocyte differentiation and matrix production, even in cells that have undergone serial passaging.

Second, 3D culture presents greater difficulties in the serial passage process, owing to the intricate methodology involved in breaking down the scaffold and harvesting the cells. Though passage in 3D model has been rarely reported, Takahashi et al. [98] utilized a 3D collagen gel culture with repeated passaging to prevent human auricular chondrocyte dedifferentiation. In the 3D culture, chondrocytes showed better suppression of collagen type I (COL1) and preservation of collagen type II (COL2) during multiplication compared to the monolayer. Tissue-engineered cartilage from 3D cells demonstrated abundant accumulation of COL2 or proteoglycan and favorable mechanical properties. It was also found that in the context of the 3D culture passaging, auricular chondrocytes exhibited a comparatively accelerated proliferation rate and a diminished expression of proteinases as opposed to nasoseptal chondrocytes [99].

Third, chondrocytes cultivated in a 3D environment may not undergo identical mechanical loading and forces as those observed in their natural cartilage habitat, thereby potentially affecting their differentiation and matrix production. The characteristics of a 3D scaffold, including its rigidity, permeability, and biological activity, substantially influence the behavior of chondrocytes and tissue growth. The task of optimizing these scaffold properties to suit particular applications and cell types remains considerable difficulties [53]. The efforts to replicate the mechanical milieu of native cartilage within a 3D culture setting still pose a formidable challenge [90]. Another strategy involves optimizing the properties of 3D scaffolds by fine-tuning factors such as mechanical strength, temperature [100], porosity, and bioactivity [55]. Applying appropriate mechanical forces to 3D chondrocyte cultures can help maintain phenotype and stimulate matrix production. It is found that dynamic compression or hydrostatic pressure can mimic the natural mechanical environment of cartilage and promote chondrogenic gene expression [101]. In addition, the concurrent utilization of hypoxic and perfusion culture techniques resulted in a notable augmentation in chondrocyte proliferation and glycosaminoglycan production [102]. Chondrocytes are naturally found in a hypoxic environment within cartilage tissue. Exposing chondrocytes to low oxygen tension (hypoxia) before or during 3D culture can enhance their proliferation and maintain their chondrogenic phenotype. Hypoxia activates hypoxia-inducible factor (HIF) pathways, which regulate various genes involved in chondrocyte survival, proliferation, and ECM synthesis [100].

2.

Fourth, the current costs of tissue-engineered cartilage are significantly higher than traditional autologous cartilage treatments due to their complexity of the production process, specialized requirements of culture system, regulatory challenges and limited clinical translation [103].

### 5.2 Alternative cell sources and comparative analysis

MSCs are widely researched and applied in clinical settings for cartilage repair and used as an alternative cell source for chondrogenesis due to their regenerative capabilities. They are easy to be isolated from adult tissues like bone marrow or adipose tissue and possess higher proliferation rate than chondrocytes [104]. However, chondrocytes produced more extracellular matrix per seeded cell than MSCs in scaffold-free cartilage discs.^107^The proteomics analysis showed that articular chondrocyte-derived cartilage contained more articular cartilage proteins, while MSC-derived cartilage had more proteins associated with cartilage hypertrophy and bone formation [105].

Compared to MSCs and chondrocytes, the induced pluripotent stem cells (iPSCs) for chondrogenesis has very high proliferation rate with extensive expansion potential without limitations [106]. They offer virtually unlimited expansion potential, providing a large number of cells for cartilage engineering [109,110].They can be generated from patient-specific cells, reducing immunorejection risks, allowing for autologous transplantation and disease modeling. Furthermore, in comparison with adult articular chondrocytes, IPSC-chondrocytes deposited two-fold more proteoglycan per cell [107]. However they also have higher risk of teratoma formation, higher cost and more time-consuming procedures [108]. The potential for genetic alterations during reprogramming limit the application of iPSCs [108,109].

Moreover, using another stem cells as chondrogenesis sources or co-culturing chondrocytes with other cell types, such as MSCs [8,92], synovium-derived stem cells (SDSCs), human embryonic stem cells (hESCs) or fibroblasts, has been found to help maintain the chondrogenic differentiation capacity in 3D culture.

The MSCs co-cultured chondrocytes have a natural chondrogenic potential that can enhance cartilage formation, suppressing hypertrophy markers in MSCs, and promoting a more stable cartilage phenotype than chondrocytes [110,111]. Recent studies have demonstrated that co-transplantation of primary chondrocytes with iPSC-derived MSCs (iMSCs) can lead to enhanced cartilage regeneration compared to using MSC-derived chondrocytes alone [112]. The iMSCs show high chondrogenic potential and are able to overcome clinical barriers associated with adult MSCs. In spite of more complex differentiation protocols, time-consuming reprogramming and differentiation processes, iMSCs may be more suitable for long-term studies or applications requiring a large number of cells or specific cell types [113]. Nevertheless, this area of research continues, and protocols for both types of cells are being refined and improved.

### 5.3 The clinical translation and regulatory landscape

As cell-based therapies for cartilage regeneration progress towards clinical application, navigating the complex landscape of clinical translation and regulatory compliance becomes paramount. Regulatory frameworks, primarily overseen by agencies like the FDA (US) and EMA (Europe), dictate stringent requirements for preclinical and clinical studies, ensuring the safety and efficacy of these novel therapies. Robust preclinical data demonstrating the absence of immunogenicity, tumorigenicity, and other adverse events are essential for regulatory approval.

Standardization and scalability present significant challenges. Developing good manufacturing practices (GMP) to ensure consistent product quality and reproducible manufacturing processes is crucial. Additionally, scalable bioreactor systems are needed for large-scale production, enabling wider access to these therapies.

Well-designed clinical trials are pivotal in assessing the long-term safety and efficacy of cell-based cartilage treatments. Patient selection, appropriate outcome measures, and extended follow-up periods are critical components of these trials. Considerations must also be given to the potential long-term effects and the durability of cartilage repair or regeneration.

Ethical considerations surrounding cell sourcing cannot be overlooked. Informed consent from donors, particularly when using embryonic or fetal tissues, is imperative. Responsible translation of research findings into clinical practice entails transparent communication of potential risks and benefits to patients and healthcare providers.

The future of cartilage regeneration lies in continuous research and development. Novel approaches, such as gene editing to enhance chondrocyte function or the use of bioprinting for precise tissue engineering, hold promise. As the field evolves, collaboration between researchers, clinicians, regulatory bodies, and industry partners will be essential to overcome remaining challenges and bring safe, effective, and accessible cell-based cartilage therapies to patients in need.

## 6 Conclusion

3D chondrocyte culture has emerged as a valuable tool for advancing tissue engineering and strategies for repairing and regenerating cartilage. This technique allows the development of in vitro models that closely mimic physiological conditions, prevent chondrocyte dedifferentiation, and enable researchers to study chondrocyte behavior in a controlled environment.

The incorporation of various signaling molecules such as growth factors and cytokines into 3D chondrocyte cultures can enhance chondrocyte growth and ECM production, ultimately leading to improved formation of cartilage tissue. Additionally, chondrocytes are often co-cultured with other cell types such as MSCs to enhance their potential for cartilage formation and promote regeneration.

However, there are limitations to 3D chondrocyte culture that must be overcome, such as challenges associated with passaging and maintenance of the chondrogenic phenotype. This requires optimization of culture conditions, including oxygen tension, nutrient supply, and mechanical stimulation.

Emerging trends in 3D chondrocyte culture include the use of bioreactor systems, decellularized matrices, and scaffold-free techniques that offer new possibilities for cartilage repair and regeneration. These advancements provide promising avenues for further research and the development of novel strategies for cartilage repair and regeneration.

In conclusion, 3D chondrocyte culture holds significant promise in the field of tissue engineering and in the development of innovative approaches for cartilage repair and regeneration. However, further research and optimization of the culture conditions are required to fully harness its potential and translate it into clinical applications.

## References

[1] Nahian A, Sapra A. (2023).

[2] Gao Y, Liu S, Huang J, Guo W, Chen J, Zhang L (2014). The ECM-cell interaction of cartilage extracellular matrix on chondrocytes. BioMed Res Int.

[3] Akkiraju H, Nohe A. (2015). Role of chondrocytes in cartilage formation, progression of osteoarthritis and cartilage regeneration. J Dev Biol.

[4] Vincent TL, McClurg O, Troeberg L. (2022). The extracellular matrix of articular cartilage controls the bioavailability of pericellular matrix-bound growth factors to drive tissue homeostasis and repair. Int J Mol Sci.

[5] Shi Y, Hu X, Cheng J, Zhang X, Zhao F, Shi W (2019). A small molecule promotes cartilage extracellular matrix generation and inhibits osteoarthritis development. Nat Commun.

[6] Fujii Y, Liu L, Yagasaki L, Inotsume M, Chiba T, Asahara H. (2022). Cartilage homeostasis and osteoarthritis. Int J Mol Sci.

[7] Zhang L, Hu J, Athanasiou KA. (2009). The role of tissue engineering in articular cartilage repair and regeneration. Crit Rev Biomed Eng.

[8] Hubka KM., Dahlin RL., Meretoja VV., Kasper FK., Mikos AG. (2014). Enhancing chondrogenic phenotype for cartilage tissue engineering: monoculture and coculture of articular chondrocytes and mesenchymal stem cells. Tissue Eng Part B.

[9] Wenger R, Hans MG, Welter JF, Solchaga LA, Sheu YR, Malemud CJ. (2006). Hydrostatic pressure increases apoptosis in cartilage-constructs produced from human osteoarthritic chondrocytes. Front Biosci.

[10] Wehland M, Steinwerth P, Aleshcheva G, Sahana J, Hemmersbach R, Lützenberg R (2020). Tissue engineering of cartilage using a random positioning machine. Int J Mol Sci.

[11] Dessau W, Vertel BM, von der Mark H, von der Mark K. (1981). Extracellular matrix formation by chondrocytes in monolayer culture. J Cell Biol.

[12] Al-Maslamani NA, Oldershaw R, Tew S, Curran J, D'Hooghe P, Yamamoto K (2022). Chondrocyte de-differentiation: biophysical cues to nuclear alterations. Cells.

[13] Tew SR, Murdoch AD, Rauchenberg RP, Hardingham TE (2008). Cellular methods in cartilage research: primary human chondrocytes in culture and chondrogenesis in human bone marrow stem cells. Methods.

[14] Charlier E, Deroyer C, Ciregia F, Malaise O, Neuville S, Plener Z (2019). Chondrocyte dedifferentiation and osteoarthritis (OA). Biochem Pharmacol.

[15] Galéra P, Rédini F, Vivien D, Bonaventure J, Penfornis H, Loyau G (1992). Effect of transforming growth factor-beta 1 (TGF-beta 1) on matrix synthesis by monolayer cultures of rabbit articular chondrocytes during the dedifferentiation process. Exp Cell Res.

[16] Mitani G, Sato M, Lee JI, Kaneshiro N, Ishihara M, Ota N (2009). The properties of bioengineered chondrocyte sheets for cartilage regeneration. BMC Biotechnol.

[17] Chiesa-Estomba CM, Hernáez-Moya R, Rodiño C, Delgado A, Fernández-Blanco G, Aldazabal J (2022). Ex vivo maturation of 3D-printed, chondrocyte-laden, polycaprolactone-based scaffolds prior to transplantation improves engineered cartilage substitute properties and integration. Cartilage.

[18] Antoni D, Burckel H, Josset E, Noel G (2015). Three-dimensional cell culture: a breakthrough in vivo. Int J Mol Sci.

[19] Shen J, Hu Z, Zhong X, Wang D, Xu L (2013). [Restoring phenotype of dedifferentiated normal nucleus pulposus cells by resveratrol]. Zhongguo Xiu Fu Chong Jian Wai Ke Za. Zhi.

[20] (2014). Mathieu M, Vigier S, Labour MN, Jorgensen C, Belamie E, Noël D, Induction of mesenchymal stem cell differentiation and cartilage formation by cross-linker-free collagen microspheres. Eur Cell Mater.

[21] Guo JF, Jourdian GW, MacCallum DK (1989). Culture and growth characteristics of chondrocytes encapsulated in alginate beads. Connect Tissue Res.

[22] Bujía J, Reitzel Sittinger M (1995). [In vitro cultivation of cartilage tissue for reconstructive surgery: effect of L(+)-lactate and glycolate on cultivated human chondrocytes]. Laryngo-Rhino-Otol.

[23] Chu CR, Coutts RD, Yoshioka M, Harwood FL, Monosov AZ, Amiel D (1995). Articular cartilage repair using allogeneic perichondrocyte-seeded biodegradable porous polylactic acid (PLA): a tissue-engineering study. J Biomed Mater Res.

[24] Ishaug-Riley SL, Okun LE, Prado G, Applegate MA, Ratcliffe A (1999). Human articular chondrocyte adhesion and proliferation on synthetic biodegradable polymer films. Biomaterials.

[25] Kapałczyńska M, Kolenda T, Przybyła W, Zajączkowska M, Teresiak A, Filas V (2018). 2D and 3D cell cultures - a comparison of different types of cancer cell cultures. Arch Med Sci.

[26] Layman DL, Sokoloff L, Miller EJ. (1972). Collagen synthesis by articular in monolayer culture. Exp Cell Res.

[27] Dessau W, Sasse J, Timpl R, Jilek F (1978). von der Mark K.Synthesis and extracellular deposition of fibronectin in chondrocyte cultures. Response to the removal of extracellular cartilage matrix. The Journal of cell biology.

[28] Peterson L, Minas T, Brittberg M, Nilsson A, Sjögren-Jansson E, Lindahl A (2000). Two- to 9-year outcome after autologous chondrocyte transplantation of the knee. Clin Orthop Relat Res.

[29] Habanjar O, Diab-Assaf M, Caldefie-Chezet F, Delort L (2021). 3D cell culture systems: tumor application, advantages, and disadvantages. Int J Mol Sci.

[30] Caron MM, Emans PJ, Coolsen MM, Voss L, Surtel DA, Cremers A (2012). Redifferentiation of dedifferentiated human articular chondrocytes: comparison of 2D and 3D cultures. Osteoarthritis Cartilage.

[31] Ghosh S, Scott AK, Seelbinder B, Barthold JE, Martin BMS, Kaonis S (2022). Dedifferentiation alters chondrocyte nuclear mechanics during in vitro culture and expansion. Biophys J.

[32] Chen Y, Yu Y, Wen Y, Chen J, Lin J, Sheng Z (2022). A high-resolution route map reveals distinct stages of chondrocyte dedifferentiation for cartilage regeneration. Bone Research.

[33] Wang X, Xue Y, Ye W, Pang J, Liu Z, Cao Y (2018). The MEK-ERK1/2 signaling pathway regulates hyaline cartilage formation and the redifferentiation of dedifferentiated chondrocytes in vitro. Am J Transl Res.

[34] Rosenzweig DH, Ou SJ, Quinn TM. (2013). P38 mitogen-activated protein kinase promotes dedifferentiation of primary articular chondrocytes in monolayer culture. J Cell Mol Med.

[35] Parreno J, Nabavi Niaki M, Andrejevic K, Jiang A, Wu PH, Kandel RA (2017). Interplay between cytoskeletal polymerization and the chondrogenic phenotype in chondrocytes passaged in monolayer culture. J Anat.

[36] Hall AC. (2019). The role of chondrocyte morphology and volume in controlling phenotype-implications for osteoarthritis, cartilage repair, and cartilage engineering. Curr Rheumatol Rep.

[37] Bush PG (2003). Hall AC,The volume and morphology of chondrocytes within non-degenerate and degenerate human articular cartilage. Osteoarthritis Cartilage.

[38] Benya PD, Padilla SR, Nimni ME (1978). Independent regulation of collagen types by chondrocytes during the loss of differentiated function in culture. Cell.

[39] Stewart MC, Saunders KM, Burton-Wurster N, Macleod JN, Stewart M.C., Saunders K.M., Burton-Wurster N., Macleod J.N. (2000). Phenotypic stability of articular chondrocytes in vitro: the effects of culture models, bone morphogenetic protein 2, and serum supplementation. J Bone Miner Res.

[40] Rosser J, Bachmann B, Jordan C, Ribitsch I, Haltmayer E, Gueltekin S (2019). Microfluidic nutrient gradient-based three-dimensional chondrocyte culture-on-a-chip as an in vitro equine arthritis model. Mater Today Bio.

[41] Benya PD, Shaffer JD (1982). Dedifferentiated chondrocytes reexpress the differentiated collagen phenotype when cultured in agarose gels. Cell.

[42] Baghaban Eslaminejad M, Taghiyar L (2009). Falahi F.Quantitative analysis of the proliferation and differentiation of rat articular chondrocytes in alginate 3D culture. Iran Biomed J.

[43] Wang CH, Tsai CH, Lin TL (2021). Liu SP.The effects of Tgfb1 and Csf3 on chondrogenic differentiation of iPS cells in 2D and 3D culture environment. Int J Mol Sci.

[44] Kanamoto T, Hikida M, Sato S, Oyama S, Tachi Y, Kuroda S (2021). Integrin α2β1 plays an important role in the interaction between human articular cartilage-derived chondrocytes and atelocollagen gel. Sci Rep.

[45] Duval K, Grover H, Han LH, Mou Y, Pegoraro AF, Fredberg J (2017). Modeling physiological events in 2D vs. 3D cell culture. Physiology.

[46] Edmondson R, Broglie JJ, Adcock AF, Yang L (2014). Three-dimensional cell culture systems and their applications in drug discovery and cell-based biosensors. Assay Drug Dev Technol.

[47] von der Mark K, Gauss V, von der Mark H, Müller P (1977). Relationship between cell shape and type of collagen synthesised as chondrocytes lose their cartilage phenotype in culture. Nature.

[48] Rim YA, Nam Y, Park N, Lee J, Park SH, Ju JH (2018). Repair potential of nonsurgically delivered induced pluripotent stem cell-derived chondrocytes in a rat osteochondral defect model. J Tissue Eng Regen Med.

[49] Akiyama H, Lyons JP, Mori-Akiyama Y, Yang X, Zhang R (2004). Zhang Z,et al. Interactions between Sox9 and beta-catenin control chondrocyte differentiation. Genes Dev.

[50] Fontoura JC, Viezzer C, Dos Santos FG, Ligabue RA, Weinlich R, Puga RD (2020). Comparison of 2D and 3D cell culture models for cell growth, gene expression and drug resistance. Mater Sci Eng C.

[51] Kudva AK, Luyten FP, Patterson J (2017). Initiating human articular chondrocyte re-differentiation in a 3D system after 2D expansion. J Mater Sci Mater Med.

[52] Ecke A, Lutter AH, Scholka J, Hansch A, Becker R (2019). Anderer U Tissue specific differentiation of human chondrocytes depends on cell microenvironment and serum selection. Cells.

[53] Li X, Chen S, Li J, Wang X, Zhang J, Kawazoe N (2016). 3D culture of chondrocytes in gelatin hydrogels with different stiffness. Polymers.

[54] Komori T (2018). Runx2, an inducer of osteoblast and chondrocyte differentiation. Histochem Cell Biol.

[55] Smeriglio P, Lai JH, Yang F, Bhutani N (2015). 3D hydrogel scaffolds for articular chondrocyte culture and cartilage generation. J Vis Exp.

[56] Foldager CB, Nielsen AB, Munir S, Ulrich-Vinther M, Søballe K, Bünger C (2011). Combined 3D and hypoxic culture improves cartilage-specific gene expression in human chondrocytes. Acta Orthop.

[57] Shoulders MD, Raines RT. (2009). Collagen structure and stability. Annu Rev Biochem.

[58] Hu D, Shan X (2017). Effects of different concentrations of type-I collagen hydrogel on the growth and differentiation of chondrocytes. Exp Ther Med.

[59] Jin GZ, Kim HW (2017). Effects of type I collagen concentration in hydrogel on the growth and phenotypic expression of rat chondrocytes. Tissue Eng Regen Med.

[60] Riacci L, Sorriento A, Ricotti L (2021). Genipin-based crosslinking of jellyfish collagen 3D hydrogels. Gels.

[61] Caliari SR, Burdick JA (2016). A practical guide to hydrogels for cell culture. Nat Methods.

[62] Liu HW, Su WT, Liu CY, Huang CC (2022). Highly organized porous gelatin-based scaffold by microfluidic 3D-foaming technology and dynamic culture for cartilage tissue engineering. Int J Mol Sci.

[63] Tsanaktsidou E, Kammona O (2022). Kiparissides C Recent developments in hyaluronic acid-based hydrogels for cartilage tissue engineering applications. Polymers.

[64] Mahapatra C, Jin GZ, Kim HW (2016). Alginate-hyaluronic acid-collagen composite hydrogel favorable for the culture of chondrocytes and their phenotype maintenance. Tissue Eng Regen Med.

[65] Wang M, Deng Z, Guo Y, Xu P (2022). Designing functional hyaluronic acid-based hydrogels for cartilage tissue engineering. Mater Today Bio.

[66] Hu X, Li D, Zhou F, Gao C (2011). Biological hydrogel synthesized from hyaluronic acid, gelatin and chondroitin sulfate by click chemistry. Acta Biomater.

[67] Park SH, Seo JY, Park JY, Ji YB, Kim K, Choi HS (2019). An injectable, click-crosslinked, cytomodulin-modified hyaluronic acid hydrogel for cartilage tissue engineering. NPG Asia Mater.

[68] Yamazaki S, Hirayama R, Ikeda Y, Iseki S, Yoda T, Ikeda MA (2023). Hyaluronic acid hydrogels support to generate integrated bone formation through endochondral ossification in vivo using mesenchymal stem cells. PLoS One.

[69] Zhu D, Trinh P, Liu E, Yang F (2023). Cell-cell interactions enhance cartilage zonal development in 3D gradient hydrogels. ACS Biomater Sci Eng.

[70] Varghese S, Hwang NS, Canver AC, Theprungsirikul P, Lin DW, Elisseeff J (2008). Chondroitin sulfate based niches for chondrogenic differentiation of mesenchymal stem cells. Matrix Biol.

[71] Buckwalter JA, Mankin HJ (1998). Articular cartilage: tissue design and chondrocyte-matrix interactions. Instr Course Lect.

[72] Wang DA, Varghese S, Sharma B, Strehin I, Fermanian S, Gorham J (2007). Multifunctional chondroitin sulphate for cartilage tissue-biomaterial integration. Nat Mater.

[73] Simson JA, Strehin IA, Allen BW, Elisseeff JH (2013). Bonding and fusion of meniscus fibrocartilage using a novel chondroitin sulfate bone marrow tissue adhesive. Tissue Eng.

[74] Ingavle GC, Dormer NH, Gehrke SH, Detamore MS (2012). Using chondroitin sulfate to improve the viability and biosynthesis of chondrocytes encapsulated in interpenetrating network (IPN) hydrogels of agarose and poly(ethylene glycol) diacrylate. J Mater Sci Mater Med.

[75] Ingavle GC, Frei AW, Gehrke SH, Detamore MS (2013). Incorporation of aggrecan in interpenetrating network hydrogels to improve cellular performance for cartilage tissue engineering. Tissue Eng.

[76] Snyder TN, Madhavan K, Intrator M, Dregalla RC, Park D (2014). A fibrin/hyaluronic acid hydrogel for the delivery of mesenchymal stem cells and potential for articular cartilage repair. J Biol Eng.

[77] Bachmann B, Spitz S, Schädl B, Teuschl AH, Redl H, Nürnberger S (2020). Stiffness matters: fine-tuned hydrogel elasticity alters chondrogenic redifferentiation. Front Bioeng Biotechnol.

[78] Mhanna R, Kashyap A, Palazzolo G, Vallmajo-Martin Q, Becher J, Möller S (2014). Chondrocyte culture in three dimensional alginate sulfate hydrogels promotes proliferation while maintaining expression of chondrogenic markers. Tissue Eng.

[79] Andersen T, Auk-Emblem P, Dornish M (2015). 3D cell culture in alginate hydrogels. Microarrays.

[80] Oliver-Ferrándiz M, Milián L, Sancho-Tello M, Martín de Llano JJ, Gisbert Roca F, Martínez-Ramos C, et al. Alginate-agarose hydrogels improve the in vitro differentiation of human dental pulp stem cells in chondrocytes. A histological study. Biomedicines 2021;9(7):834. 10.3390/biomedicines9070834 PMC8301309 34356898

[81] Miao Z, Lu Z, Wu H, Liu H, Li M, Lei D (2018). Collagen, agarose, alginate, and Matrigel hydrogels as cell substrates for culture of chondrocytes in vitro: a comparative study. J Cell Biochem.

[82] Fu Y, Zoetebier B, Both S, Dijkstra PJ, Karperien M (2021). Engineering of optimized hydrogel formulations for cartilage repair. Polymers.

[83] Jin R, Teixeira LS, Dijkstra PJ, van Blitterswijk CA, Karperien M, Feijen J (2010). Enzymatically-crosslinked injectable hydrogels based on biomimetic dextran-hyaluronic acid conjugates for cartilage tissue engineering. Biomaterials.

[84] Ahmadian E, Eftekhari A, Janas D, Vahedi P (2023). Nanofiber scaffolds based on extracellular matrix for articular cartilage engineering: a perspective. Nanotheranostics.

[85] Kim YS, Majid M, Melchiorri AJ, Mikos AG (2019). Applications of decellularized extracellular matrix in bone and cartilage tissue engineering. Bioeng Transl Med.

[86] Watanabe T, Asawa Y, Watanabe M, Okubo R, Nio M, Takato T (2020). The usefulness of the decellularized matrix from three-dimensional regenerative cartilage as a scaffold material. Regen Ther.

[87] Cooper SM, Rainbow RS (2022). The developing field of scaffold-free tissue engineering for articular cartilage repair. Tissue Eng Part B.

[88] Martinez I, Elvenes J, Olsen R, Bertheussen K, Johansen O (2008). Redifferentiation of in vitro expanded adult articular chondrocytes by combining the hanging-drop cultivation method with hypoxic environment. Cell Transplant.

[89] Ariyoshi W, Usui M, Sano K, Kawano A, Okinaga T, Nakashima K (2020). 3D spheroid culture models for chondrocytes using polyethylene glycol-coated microfabricated chip. Biomed Res.

[90] Mellor LF, Baker TL, Brown RJ, Catlin LW, Oxford JT (2014). Optimal 3D culture of primary articular chondrocytes for use in the rotating wall vessel bioreactor. Aviat Space Environ Med.

[91] Fu L, Li P, Li H, Gao C, Yang Z, Zhao T (2021). The application of bioreactors for cartilage tissue engineering: advances, limitations, and future perspectives. Stem Cell Int.

[92] Levorson EJ, Santoro M, Kasper FK, Mikos AG (2014). Direct and indirect co-culture of chondrocytes and mesenchymal stem cells for the generation of polymer/extracellular matrix hybrid constructs. Acta Biomater.

[93] Tangyuenyong S, Kongdang P, Sirikaew N, Ongchai S (2022). First study on the effect of transforming growth factor beta 1 and insulin-like growth factor 1 on the chondrogenesis of elephant articular chondrocytes in a scaffold-based 3D culture model. Vet World.

[94] Xiao J, Chen X, Xu L, Zhang Y, Yin Q, Wang F (2014). PDGF regulates chondrocyte proliferation through activation of the GIT1- and PLCγ1-mediated ERK1/2 signaling pathway. Mol Med Rep.

[95] Kieswetter K, Schwartz Z, Alderete M, Dean DD, Boyan BD (1997). Platelet derived growth factor stimulates chondrocyte proliferation ut prevents endochondral maturation. Endocrine.

[96] Zhao GZ, Zhang LQ, Liu Y, Fang J, Li HZ, Gao KH (2016). Effects of platelet-derived growth factor on chondrocyte proliferation, migration and apoptosis via regulation of GIT1 expression. Mol Med Rep.

[97] Al-Masawa ME, Wan Kamarul Zaman WS, Chua KH (2020). Biosafety evaluation of culture-expanded human chondrocytes with growth factor cocktail: a preclinical study. Sci Rep.

[98] Takahashi T, Ogasawara T, Asawa Y, Mori Y, Uchinuma E, Takato T (2007). Three-dimensional microenvironments retain chondrocyte phenotypes during proliferation culture. Tissue Eng.

[99] Asawa Y, Ogasawara T, Takahashi T, Yamaoka H, Nishizawa S, Matsudaira K (2009). Aptitude of auricular and nasoseptal chondrocytes cultured under a monolayer or three-dimensional condition for cartilage tissue engineering. Tissue Eng.

[100] Hu X, Zhang W, Li X, Zhong D, Li Y, Li J (2021). Strategies to modulate the redifferentiation of chondrocytes. Front Bioeng Biotechnol.

[101] Anderson DE, Johnstone B (2017). Dynamic mechanical compression of chondrocytes for tissue engineering: a critical review. Front Bioeng Biotechnol.

[102] Dahlin RL, Meretoja VV, Ni M, Kasper FK, Mikos AG (2013). Hypoxia and flow perfusion modulate proliferation and gene expression of articular chondrocytes on porous scaffolds. AIChE J.

[103] Francis SL, Di Bella C, Wallace GG, Choong PFM (2018). Cartilage tissue engineering using stem cells and bioprinting technology-barriers to clinical translation. Front Surg.

[104] Chen H, Tan XN, Hu S, Liu RQ, Peng LH, Li YM (2021). Molecular mechanisms of chondrocyte proliferation and differentiation. Front Cell Dev Biol.

[105] Frerker N, Karlsen TA, Stensland M, Nyman TA, Rayner S, Brinchmann JE (2023). Comparison between articular chondrocytes and mesenchymal stromal cells for the production of articular cartilage implants. Front Bioeng Biotechnol.

[106] Lee M-S, Sivapatham A, Leiferman EM, Jiao H, Lu Y, Nemke BW (2023). Autologous iPSC- and MSC-derived chondrocyte implants for cartilage repair in a miniature pig model. bioRxiv.

[107] Diederichs S, Klampfleuthner FAM, Moradi B, Richter W (2019). Chondral differentiation of induced pluripotent stem cells without progression into the endochondral pathway. Front Cell Dev Biol.

[108] Thanaskody K, Jusop AS, Tye GJ, Wan Kamarul Zaman WS, Dass SA, Nordin F (2022). MSCs vs. iPSCs: potential in therapeutic applications. Front Cell Dev Biol.

[109] Lach MS, Rosochowicz MA, Richter M, Jagiełło I, Suchorska WM, Trzeciak T (2022). The induced pluripotent stem cells in articular cartilage regeneration and disease modelling: are we ready for their clinical use?. Cells.

[110] Somoza RA, Welter JF, Correa D, Caplan AI (2014). Chondrogenic differentiation of mesenchymal stem cells: challenges and unfulfilled expectations. Tissue Eng Part B.

[111] Zuo Q, Cui W, Liu F, Wang Q, Chen Z, Fan W (2013). Co-cultivated mesenchymal stem cells support chondrocytic differentiation of articular chondrocytes. Int Orthop.

[112] Kim IG, Park SA, Lee SH, Choi JS, Cho H, Lee SJ (2020). Transplantation of a 3D-printed tracheal graft combined with iPS cell-derived MSCs and chondrocytes. Sci Rep.

[113] Khan NM, Diaz-Hernandez ME, Drissi H (2023). Differentiation of human induced pluripotent stem cells (iPSCs)-derived mesenchymal progenitors into chondrocytes. Bio Protoc.

